# Simultaneous ipsilateral proximal interphalangeal and metacarpophalangeal dislocation of the fifth phalanx: A case report

**DOI:** 10.1186/1757-1626-1-208

**Published:** 2008-10-03

**Authors:** Koray Unay, Korhan Ozkan, Ender Ugutmen, Feyza Unlu Ozkan, Abdullah Eren, Kerem Bilsel

**Affiliations:** 1 Goztepe Research and Training Hospital, Department of Orthopaedics and Traumatology, Istanbul, Turkey; 2 Fatih Sultan Mehmet Research and Training Hospital, Physical Theraphy and Rehabilitation, Istanbul, Turkey; 3 Istanbul University, School of Medicine, Department of Orthopaedics and Traumatology, Istanbul, Turkey

## Abstract

**Introduction:**

There is no case of simultaneous ipsilateral proximal interphalangeal and metacarpophalangeal dislocation of a finger in the literature.

**Case presentation:**

A 61 years old male patient sustained an ipsilateral dorsal dislocation of the PIP joint of his fifth finger and dorsal dislocation of the metacarpophalangeal joint. Closed reduction of proximal interphalangeal joint was achieved while open reduction of the metacarpophalangeal joint was carried out.

**Conclusion:**

The single most important element preventing reduction of the metacarpophalangeal joint was an interposition of the volar plate between proximal end of the phalanx and the head of the metacarpal.

## Introduction

In this report we aimed to investigate and discuss an extremely rare injury that is bipolar dislocation of the fifth proximal phalanx due to a fall on hand. Although, simultaneous dislocation of metacarpophalangeal and carpometacarpal joints of the same digit had been previously reported, we did not find any case of simultaneous ipsilateral proximal interphalangeal and metacarpophalangeal dislocation of a finger in the English speaking literature.

## Case presentation

**Figure 1:**
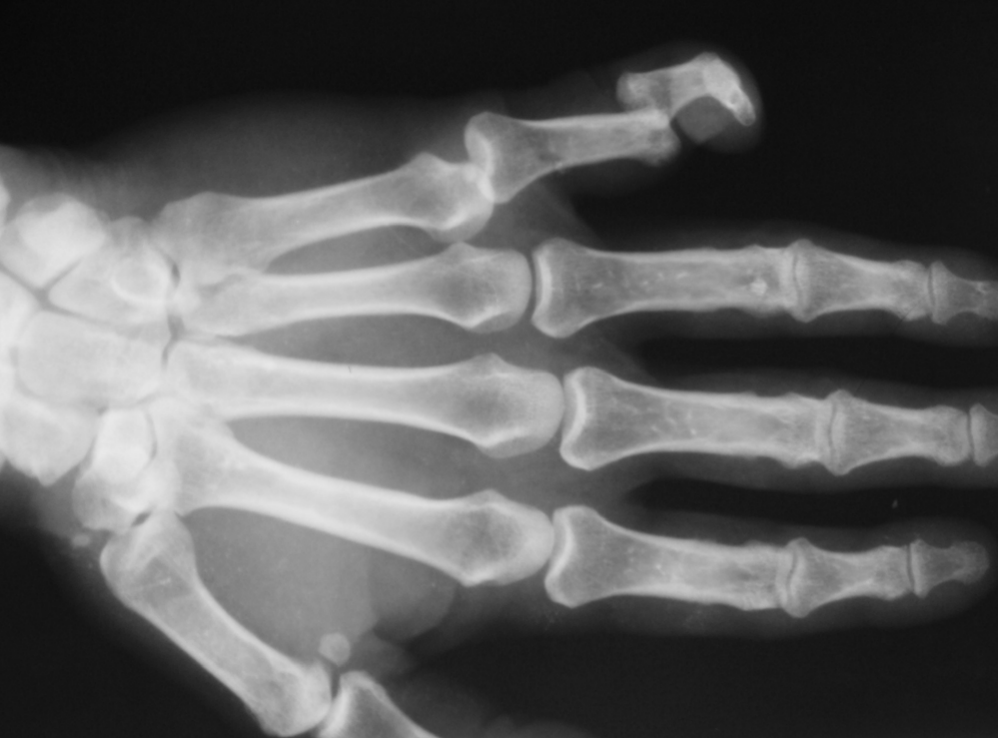
Bipolar dislocation of fifth phalanx.

A 61 years old male patient fell from a height and sustained an ipsilateral dorsal dislocation of the PIP joint of his fifth finger and dorsal dislocation of the metacarpophalangeal joint. X ray examination of the finger revealed no fracture and closed reduction with wrist block was attempted (Figure 1). Closed reduction of the fifth finger was achieved but metacarpophalangeal joint remained dislocated so open reduction was carried out. We preferred volar approach. An incision in the proximal palmar crease is extended along the mid ulnar aspect of the little finger.

**Figure 2:**
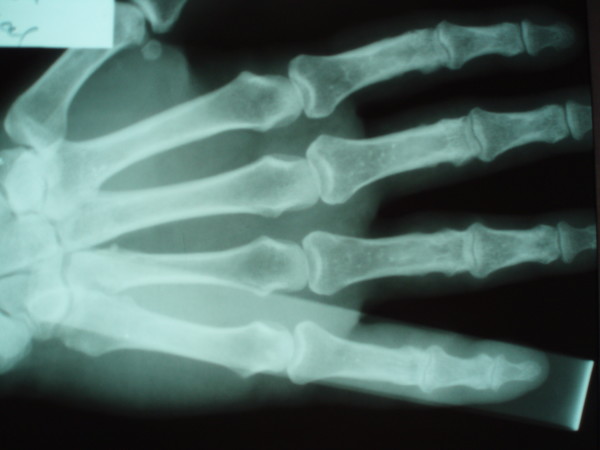
AP roentgenogram after surgery.

**Figure 3:**
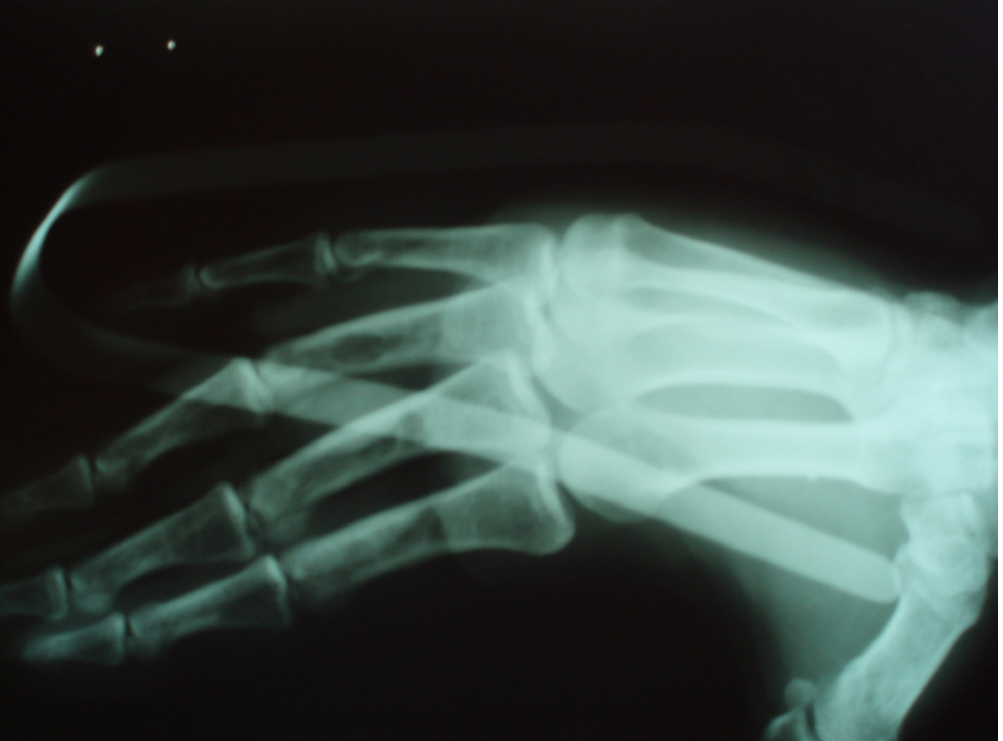
Lateral roentgenogram after surgery.

After the incision of the skin and palmar fascia metacarpal head was seen lying between the flexor tendon on radial side and ulnar digital nerve and artery on the ulnar side. These structures were retracted to provide better exposure of the joint. The single most important element preventing reduction was the interposition of the volar plate between the base of the proximal phalanx and the head of the metacarpal. The plate was found to be torn from its proximal attachment and wedged dorsal to exposed metacarpal head. A short longitudinal incision was made to split the volar plate and then volar plate was easily extricated with reduction of the joint (Figure 2 and 3).

## Discussion

We did not find any case about a dorsal dislocation of an ipsilateral proximal interphalangeal and metacarpophalangeal joint (fifth phalanx dislocation) in the English speaking literature. Irreducible metacarpophalangeal dislocations are extremely rare injuries with buttonholing of the metacarpal head into the palm [1-3].

The most important element preventing reduction is an interposition of the volar plate between the base of the proximal phalanx and the head of metacarpal. In this case report, taking the advantage of a direct access to the metacarpal head we had preferred volar approach for an open reduction of a complex metacarpophalangeal dislocation.

To us, volar approach also has the advantage of an evaluating the integrity of the neurovascular bundle (digital nerve and artery) which are tented tightly over the metacarpal head.

## Conclusion

Although, simultaneous dislocation of metacarpophalangeal and carpometacarpal joints of the same digit had been previously reported [2], simultaneous proximal interphalangeal and metacarpophalangeal dislocations of the same digit have not been discussed in the English literature and the patient consulting to the emergency unit with a trauma to his hand should be evaluated carefully for possible bipolar dislocation of fifth phalanx.

## Abbreviations

PIP: Proximal interphalangeal.

## Competing interests

The authors declare that they have no competing interests.

## Authors' contributions

KU, KO and EU contributed to conception and design, carried out the literature research, manuscript preparation and manuscript review. FUO was involved with the case and writing of themanuscript. AE revised the manuscript for important intellectual content. KB supervised the writing and the general management of the patient.

## Consent

Written consent was obtained from the patient for publication of the study.
